# In-depth LC-MS and *in-vitro* studies of a triterpenoid saponin capilliposide-A metabolism modulation in gut microbiota of mice

**DOI:** 10.3389/fphar.2024.1361643

**Published:** 2024-03-14

**Authors:** Huan Zhao, Xueli Hu, Shenghong Guan, Jinhong Cai, Xiaohan Li, Jiaxi Fang, Bo Lin, Wei Zhu, Jingkui Tian, Juan Jin, Qiang He, Xiaoyong Zhang

**Affiliations:** ^1^ Department of Nephrology, Urology and Nephrology Center, Zhejiang Provincial People’s Hospital, Affiliated People’s Hospital, Hangzhou Medical College, Hangzhou, China; ^2^ Key Laboratory for Molecular Medicine and Chinese Medicine Preparations, Hangzhou Institute of Medicine (HIM), Chinese Academy of Sciences, Hangzhou, China; ^3^ Department of Nephrology, The First Affiliated Hospital of Zhejiang Chinese Medical University (Zhejiang Provincial Hospital of Traditional Chinese Medicine), Hangzhou, China

**Keywords:** gut microbiota, metabolite, triterpenoid, saponin, LC-MS, capilliposide A, mice

## Abstract

**Introduction:** Some herbal ingredients can reshape the composition of the gut microbiome as well as its metabolites. At the same time, the gut microbiota can also affect drug metabolism. A large number of studies have reported that saponins are biotransformed under the action of intestinal microorganisms to improve drug efficacy and bioavailability. Capilliposide A is a triterpenoid saponin, which is derived from Lysimachia *capillipes* Hemsl. CPS-A has anti-inflammatory pharmacological activity, but the substance basis *in vivo* is unknown at present, so studies on the interaction between intestinal microorganisms and CPS-A may clarify the pharmacodynamic substance basis of CPS-A.

**Methods:** This study established a colitis mouse model, collected sterile feces from normal mice and colitis mice, and incubated CPS-A with two different intestinal flora *in vitro*. Based on LC-MS, the metabolic process of CPS-A mediated by intestinal microbes and the intervention effect of CPS-A on intestinal microbiome derived metabolites were studied.

**Results:** The results of experiments indicate that intestinal microorganisms can mediate the biotransformation of CPS-A and metabolize it into corresponding deglycosylation products, thereby promoting its drug effect. Not only that, CPS-A can also promote metabolites such as Deoxycholic acid, Histamine, 3-Hydroxytridecanoic acid, and Indole-3-acetic acid in the intestinal microbiota of mice with colitis. This may result in anti-colitis effects. CPS-A mainly involved in metabolic pathways such as azathioprine and mercaptopurine, which may also have beneficial or adverse effects.

**Discussion:** This study on the interaction between CPS-A and microbiota provides a new idea for the study of traditional Chinese medicine with poor oral bioavailability. The regulatory effect of CPS-A on the metabolites of intestinal flora in colitis mice was also found. It laid a foundation for exploring the mechanism of action of saponins on colitis mice.

## Introduction

Numerous natural products and their derived compounds have been extensively studied for their ability to treat diseases (Xie et al., 2019; Muhammad et al., 2021a; Muhammad et al., 2021b; Abduljawad et al., 2022). Saponins have potential anti-inflammatory properties and can promote immune homeostasis in various diseases (Li et al., 2014; Shah et al., 2014; Liu et al., 2016; Khan et al., 2022). However, due to the poor membrane permeability, the bioavailability of saponins is exceptionally low, and the substance basis of *in vivo* utilization is not clear. This also limits the further study of saponins to a certain extent. Capilliposide A is a triterpenoid saponin that is obtained from *Lysimachia capillipes* Hemsl., have been documented to exhibit good anti-inflammatory, anti-tumor, and anti-angiogenic properties (Li et al., 2022; Li et al., 2023). It is well known that intestinal microflora plays an important role in the metabolism of compounds administered orally or excreted into bile. Studies have shown that intestinal flora can biotransform flavonoids *in vitro*, and intestinal flora Proteobacteria can promote the bioavailability of flavonoids. Baicalin, papillalein A and baicalin in scutellaria baicalin were converted into more absorbable baicalin, papillalein A and baicalin (Feng et al., 2018). At the same time, pharmacokinetic studies have shown that many intestinal bacteria can convert large polar saponins into small polar compounds, so that they can quickly accumulate to the required blood concentration. For example, ginsenoside CK and ginsenoside Rg3 were obtained under intestinal flora metabolism of American ginseng, and these compounds showed better anti-inflammatory bioactivity (Wang et al., 2018). The conversion availability of the same ingredient was different in different species of intestinal bacteria. Their components are inevitably brought into contact with intestinal microflora in the alimentary tract and are metabolized by intestinal microflora before absorption from the intestinal tract into the blood (Choi et al., 2011; Crow, 2011). Thus, intestinal microflora has extensively been used for the *in vitro* metabolic study of natural products. Although the host itself lacks enzymes related to the glycoside linkage of the hydrolysis of saponins, the microbiota in the host gastrointestinal tract can secrete hydrolase to mediate the production of related secondary metabolites from saponins (Wan et al., 2013). The saponins are mainly converted into smaller molecules by sugar hydrolysis reaction under the action of intestinal microorganisms, so as to improve bioavailability and enhance drug action (Chen et al., 2016). However, the biotransformation process of CPS-A under the action of intestinal microorganisms has not yet been studied.

In addition, saponins have a relatively obvious regulatory effect on intestinal microorganisms, resulting in the generation of corresponding small molecule metabolites. These metabolites further perform many biological functions. A large number of experiments have proved that saponins can regulate the intestinal barrier and improve inflammation by regulating intestinal microbes and their source metabolites, so as to treat UC (Ananthakrishnan, 2015). Recently, A study has shown that berberine demonstrates an anti-inflammatory action and safeguards the integrity of the intestinal barrier through its interaction with the intestinal flora and regulation of associated metabolites (Jing et al., 2021). Protopanaxatriol saponin has the potential to reduce the metabolic dysfunction through the restoration of abnormal changes in 29 metabolites and the regulation of eleven metabolic pathways (Wu et al., 2022). Therefore, traditional Chinese medicine may play a biological function by regulating the relative abundance of intestinal microbial communities and thereby regulating the expression level of microbe-derived metabolites (Gao et al., 2018). At present, it has not been studied whether CPS-A has a regulatory effect on intestinal microbial-derived metabolites. Therefore, it is important to study the interaction between CPS-A and gut microbiome for elucidating the material basis of CPS-A.

The imbalance of intestinal flora is closely related to gastrointestinal inflammatory diseases and may determine the severity of intestinal inflammation (Zhang et al., 2021). In 2023, the global prevalence of UC was estimated to be around 5 million cases, and there is a rising incidence of the disease worldwide (Le Berre et al., 2023). Significantly, the elevated risk of developing colon cancer that is associated with UC has garnered considerable attention. Alternatively, evidence-based approach based on modern scientific techniques (e.g., systems biology-based ‘omics technologies) and comparative effectiveness research approach are regarded as valid strategies for exploring traditional chinese medicine (Wang et al., 2015). Therefore, in this paper, the incubation system of intestinal microorganisms *in vitro* was applied to study the metabolic material basis of CPS-A under the action of intestinal microorganisms. At the same time, metabolomics technology based on LMS was applied to study the disturbance of small molecule metabolites after CPS-A interfered with intestinal microorganisms of normal and colitis mice, so as to clarify the interaction between intestinal microorganisms and CPS-A.

## Materials and methods

### Chemicals and reagents

**FIGURE 1:**
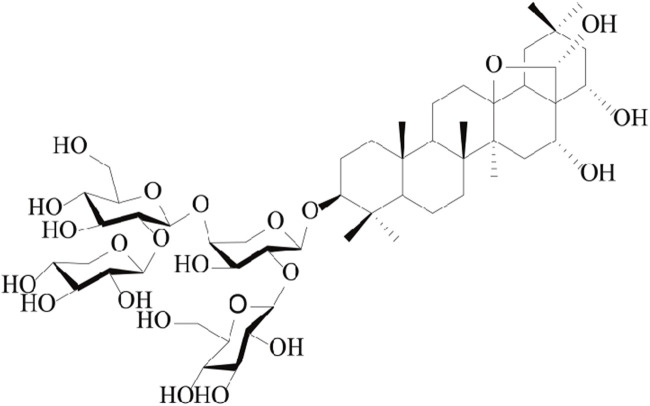
Chemical structure of CPS-A (Li et al., 2023).

The modified GAM medium was purchased from Qingdao Haibo Biotechnology Company. DMSO was purchased in Aladdin, Shanghai. The anaerobic production bag was purchased from MGC in Japan. Formic acid was obtained from Shanghai McLean. Methanol and acetonitrile were purchased from TEDIA USA. The deionized water is supplied by Cascade pure water meter of PALL Company. Capilliposide A was obtained from Professor Tian Jingkui, Institute of Basic Medical Oncology, Chinese Academy of Sciences (Zhejiang, China). The molecular structure of CPS-A is shown in Figure 1.

### Construction of *in vitro* incubation system for intestinal microorganisms

The experimental animals were purchased from the Institute of Basic Medicine and Cancer, Chinese Academy of Sciences. Healthy male C57BL/6 mice, SPF grade, weight (20 ± 2) g, all animals were kept in an SPF environment with alternating light and dark, constant temperature and humidity, free diet, and free drinking water. The animal experiment was carried out after 1 week of adaptive feeding. The animal experiment followed GB/T 35892-2018 experimental animal welfare ethics, which was approved by the Institute of Basic Medicine and Cancer of Chinese Academy of Sciences. The experimental process reduced the pain of mice as much as possible. A DSS-induced colitis mouse model was established. Mice were randomly divided into two groups (n = 6) as follows: Control, Model. Control group was given 0.2 mL CMC-Na-H2O (0.5:99.5, w/v) solution in the morning and evening, while Model group was given 0.2 mL DSS solution (400 mg/kg) and 0.2 mL CMC-Na-H2O (0.5:99.5, w/v). In this process, the weight, feeding and fecal occult blood of mice were recorded every day, and the success of colitis modeling was evaluated. After 5 days, feces of each group were collected aseptically for follow-up experiment. The mice were killed after stool collection and colon length and histopathological sections were measured to further evaluate the model. The preparation of intestinal flora in mice was based on literature reports with some adjustments (Javdan et al., 2020). 3 g of fresh feces were collected from normal mice and colitis mice, and placed into a sterile Ep tube, and 30 mL of sterile pre-cooled PBS solution was rapidly added, and after swirling for 1 min, ultrasound was carried out in a water bath at 4°C for 15 min, and centrifuged at 4°C and 100 g for 30 min, and the supernate was taken to obtain the fecal bacterial solution of mice. The modified GAM medium was weighed to about 54 g, heated and dissolved in 900 mL ultra-pure water, and autoclaved at 120°C for 20 min to obtain the microbial medium. The prepared bacterial solution was added to 200 mL mGAM medium and incubated at 37°C and 120 rpm for 48 h. The obtained bacterial solution was centrifuged at 4°C and 1,400 g for 10 min, then precipitated, washed twice with sterile pre-cooled PBS solution, and then added 40 mL mGAM medium to obtain the required intestinal microbial bacterial solution by suspension.

### Preparation of drug administration solution, sample grouping and treatment

A certain amount of CPS-A was divided into 50 mL of the centrifugation tube, and the bacterial solution was added to make the final concentration 1.0 mg/mL (0.5% DMSO was added to help dissolve). Shake well and incubate in anaerobic production bag (containing oxygen indicator) at 37°C and 120 rpm. Make 3 copies of each incubation experiment in parallel. At 0, 6, 12, 24, 36, and 48 h, 5 mL of mixed samples were taken and placed in 40 mL acetonitrile, and after being shaken and vorticed well, the supernatant was taken at 4°C and centrifuged at 17,000 g for 10 min. The supernatant was retained at 4°C for future use. The experiment was divided into 4 groups: normal mice and colitis mice fecal incubation system and 2 kinds of incubation system after adding CPS-A.

### LC-MS conditions for identification of metabolites

200 µL supernatant was taken from each time point and mixed, placed at 4°C for vacuum drying, then added with 200 µL 70% acetonitrile waters (7:3, v/v) for 30min by on-ice ultrasound, centrifuged at 4°C at 12,000 rpm for 20 min, and then tested by mass spectrometry. Samples were analyzed using ultra-performance liquid chromatography (UPLC) coupled with a Thermos Scientific Orbitrap 120 mass spectrometer. acquisition parameters: spray voltage, −2,500 V; Sheath gas (Arb), 50; Aux gas (Arb), 10; Sweep gas (Arb),1; Ion transfer tube temperature, 300°C; Vaporizer temperature, 350°C; orbitrap resolution,60000, scan range, 200-1,500; Dynamic exclusion on; HCD collision energies, 30, 50, 70 eV. Chromatographic column to Welch UPLC C18 (2.1*50 mm*1.7 um, Waters Corporation), column temperature 45°C; Mobile phase A: water (0.1% formic acid); Mobile phase B: acetonitrile; The flow rate of 0.3 mL/min. Gradient elution conditions:15% B (0–2 min), 30% B (2–3 min), 45% B (3–10 min), 60% B (25–28 min), 95% B (28–32 min), 15% B (32–35 min) (Cheng, 2015).

### LC-MS conditions for identification of fecal metabolomics

The detection methods of liquid chromatography refer to the methods developed in the literature (Zhang et al., 2018). The chromatography was performed on ACQUITY UPLC HSS T3 (2.1*50 mm*1.7 um, Waters Corporation) column at 40°C with the mobile phase ratio of A (0.1% formic acid water) to B (acetonitrile) at the flow rate of 0.35 mL/min. The sample size was 10 µL. Instrument and parameter Settings as above. Gradient elution conditions: 5% B (0–3 min), 20% B (3–5 min), 40% B (5–9 min), 60% B (9–16 min), 65% B (16–18 min),80% B (18–21 min),95% B (21–23 min),5% B (23–25 min).

### Data analysis

LC-MS data analysis was performed using the Compound discover version 3.2 (Thermo Fisher Scientific Inc). The statistical significance of differences between two groups was analyzed by Student’s t-test. The differences between multiple groups were analyzed by one-way ANOVA followed by Dunnett’s test using GraphPad v8.0. A value of *p* < 0.05 or *p* < 0.01 was defined as statistically significant.

## Results

### Colitis model was successfully induced by sodium dextran sulfate

**FIGURE 2:**
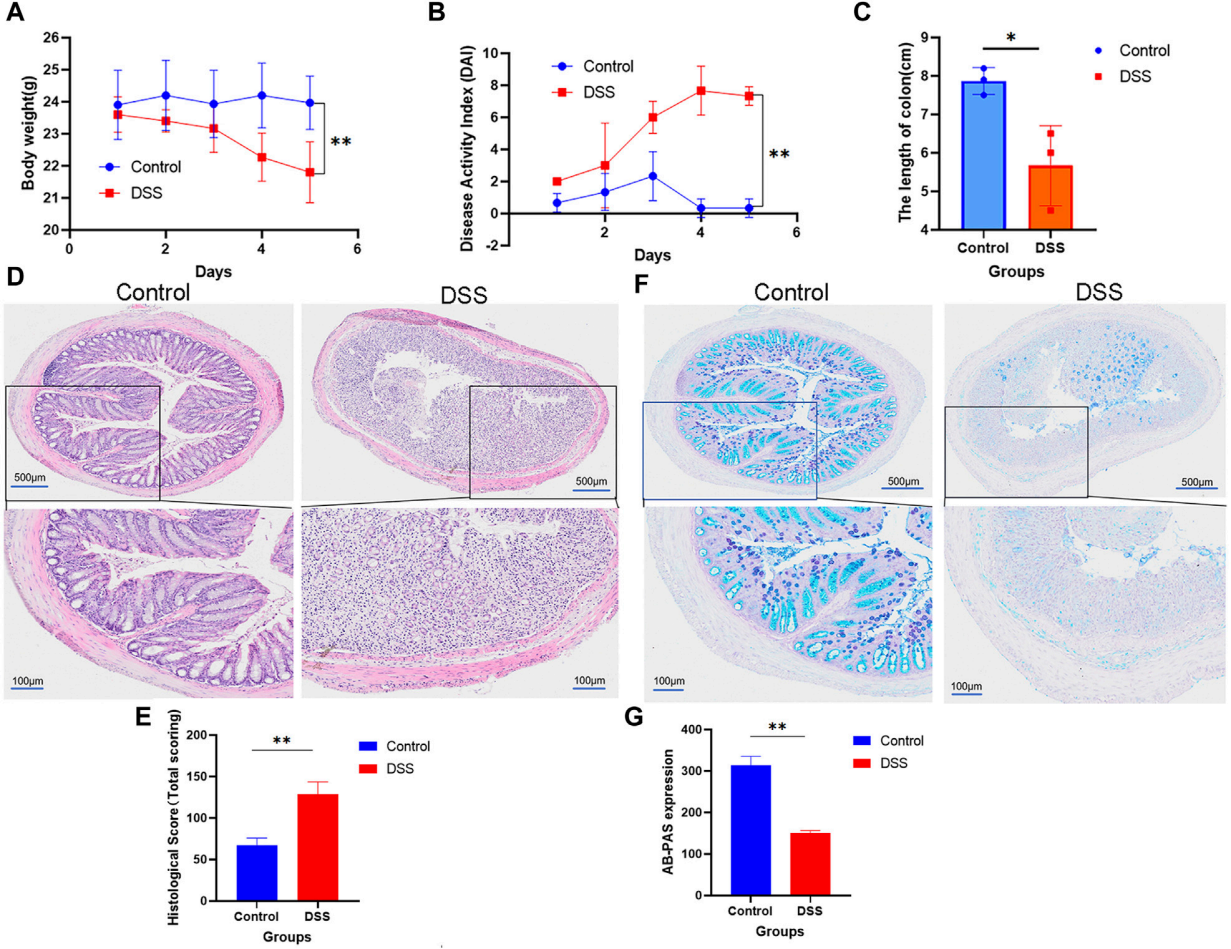
Mice with normal colitis were modulated to reserve feces for subsequent *in vitro* incubation experiments. **(A)** Weight; **(B)** DAI; **(C)** The length of colon; **(D)** HE is staining; **(E)** Statistical chart of HE scores (n = 3); **(F)** AB-PAS staining. **(G)** Statistical chart of AB-PAS (n = 3); Values are expressed as mean ± SD. *p < 0.05, * *p < 0.01 compared with DSS group.

In order to provide initial materials for the incubation system between CPS-A and intestinal bacteria populations, our study constructed normal group and colitis group models in mice, and stool collection was performed after successful model construction. Of note, mice in the DSS induced colitis group lost significantly more weight than normal mice without intervention. (Figure 2A). As shown in Figures 2B, C, DAI scores in the colitis group were significantly higher and colon length was shorter than control group. Histological assessment using hematoxylin and eosin (H&E) staining demonstrated that the sections of colonic tissue of the model group displayed significant inflammatory cell accumulation, loss of goblet and epithelial cells, creation of crypt abscesses, thickening, and separation of the muscle layer, and even the presence of ulcers (Figure 2D). Furthermore, histopathological examination revealed evident colonic tissue damage (Figure 2E). AB-PAS staining findings revealed a substantial reduction in goblet cells in the colons of mice subjected to DSS treatment. (Figures 2F, G). The significant changes of these indications indicate the successful construction of the DSS induced colitis model.

### Identification of *in vitro* metabolites of CPS-A mediated by intestinal microbes

The reaction system of intestinal microorganisms *in vitro* was detected by UPLC-MS. The fragmentation and chromatographic behavior of CPS-A were studied prior to analyzing its metabolites. CPS-A eluted at a retention time (Rt) of 5.640 min and was detected at m/z 1,077.5448. The specific ions observed were due to the loss of water and sugar components. The [M-H]^−1^ ion at m/z 1,077.5488 broke up into the following product ions, respectively: m/z 1,060.5407,1059.5393, 946.5093, 945.5066, and 927.4967 (Supplementary Figure S1). The dominant ion at 1,059.5393 can be attributed to the neutral H2O loss. Subsequently, it underwent a loss of 132 Da (Xylose), resulting in a transition to m/z 927.4967. Furthermore, the loss of xylose (Xyl) resulted in the formation of the characteristic ions observed at m/z 945.5066 in the spectrum. Previous studies have detected the metabolic status of CPS-A in plasma, feces, and urine of mice, and found that CPS-A was mainly transformed in feces (Li et al., 2023). Therefore, to confirm this result, we collected mouse feces and incubated them with CPS-A *in vitro*.

M1 showed the ion at m/z 1,059.5302, decreasing 18 Da from M0, and obtained its peak elution at 8.113 min. The results showed that one H2O was lost from CPS-A. The position of dehydration caused by multiple hydroxyl groups in the CPS-A structure cannot be determined.

M2 showed the ion at m/z 1,091.5719 with a retention time of 7.691 min, which was 15 Da higher than that of CPS-A. The product ion at m/z 959.5177 was formed after losing xylose.

M3 appeared on the peak at 6.506 min and provided the ion with a mass of m/z 621.3986, which was 456 Da less than CPS-A. The results showed that one xylose and two glucoses were lost from CPS-A. A prominent ion at m/z 603.3911 was losing H2O. Therefore, M3 was determined.

M4 was observed at 6.182 min and revealed the molecular ion at m/z 915.4984, which has a molecular weight 162 Da lower than that of the parent. According to the information on product ions, it indicated that M4 lost glucose from the parent. Two prominent ions at m/z 897.4862 and 783.4573 were losing H2O and xylose. Hence, it was identified.

M5 was identified at m/z 765.4428 in the negative mode with an retention time of 11.735 min. The mass value was 312 Da lower than that of M0, indicating that M5 had lost xylose, glucose and H2O. Consequently, M5 was identified.

**FIGURE 3:**
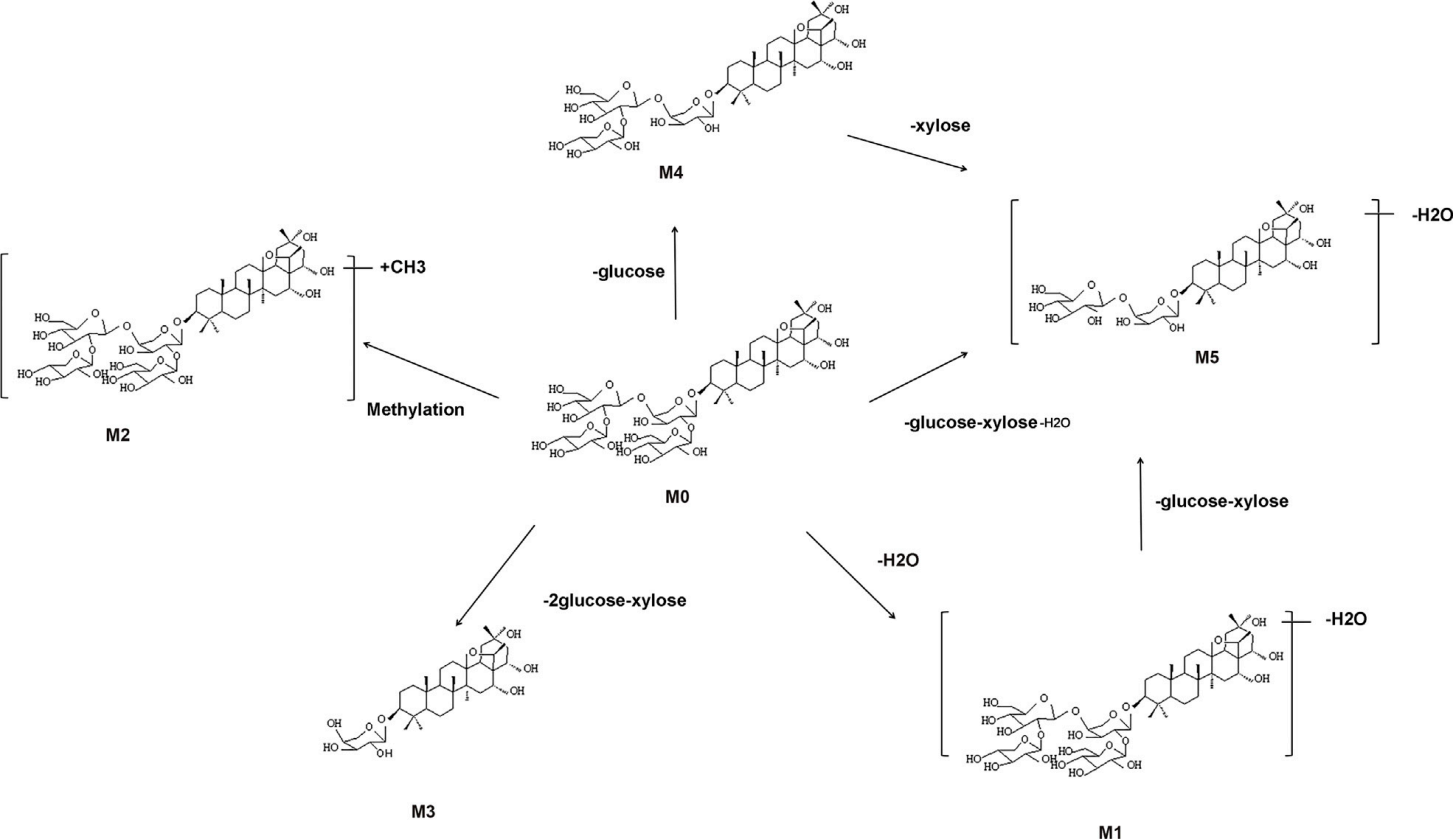
Possible metabolic pathways of CPS-A *in vitro*.

**FIGURE 4:**
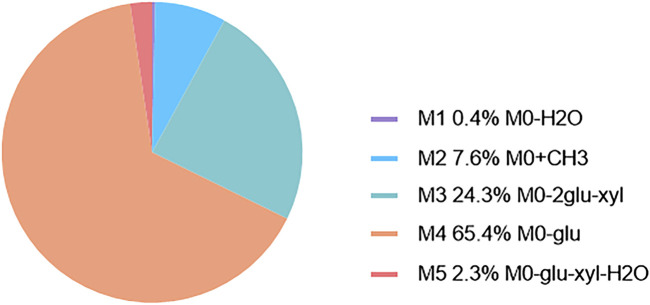
Abundance map of *in vitro* metabolites of CPS-A mediated by gut microbes.

The metabolic pathways of CPS-A *in vitro* were proposed in Figure 3. Five metabolites derived from the parent compound were shown in Table 1. The results showed that CPS-A was metabolized into the corresponding products by the transformation methods of deglycosylation, methylation, dehydration under the action of intestinal flora. Through the abundance analysis of the peak area of extracted ions, it was found that CPS-A mainly underwent deglycosylation reactions under the action of intestinal microorganisms, such as CPS-A-Glu (65.4%), CPS-A-2Glu-xyl (24.3%) and CPS-A-xyl-Glu-H2O (2.3%). As shown in Figure 4, The product abundance accounted for 92% of the total product abundance of CPS-A.

**TABLE 1:** Identification of *in vitro* metabolites of CPS-A.

Metabolites	Composition	Formula	m/z	Error (ppm)	Calc. MW	Adduct	R.T. (min)	Peak area
M1	Loss of H2O	C53H86O24	1,105.54370	0.07	1,106.55098	M + FA-H	8.113	1461173
M2	Methylation	C54H90O25	1,123.55327	−0.19	1,124.56055	M + FA-H	7.691	31135129
M3	Loss of 2 glucose and xylose	C36H60O11	667.40710	1.22	668.41438	M + FA-H	6.506	99254804
M4	Loss of glucose	C47H78O20	961.50108	−3.27	962.50836	M + FA-H	6.482	267148328
M5	Loss of glucose, xylose and H2O	C41H66O13	765.44320	0.17	766.45047	M-H	11.735	9576370

### Metabolic changes of normal mice intestinal flora after CPS-A intervention

**FIGURE 5:**
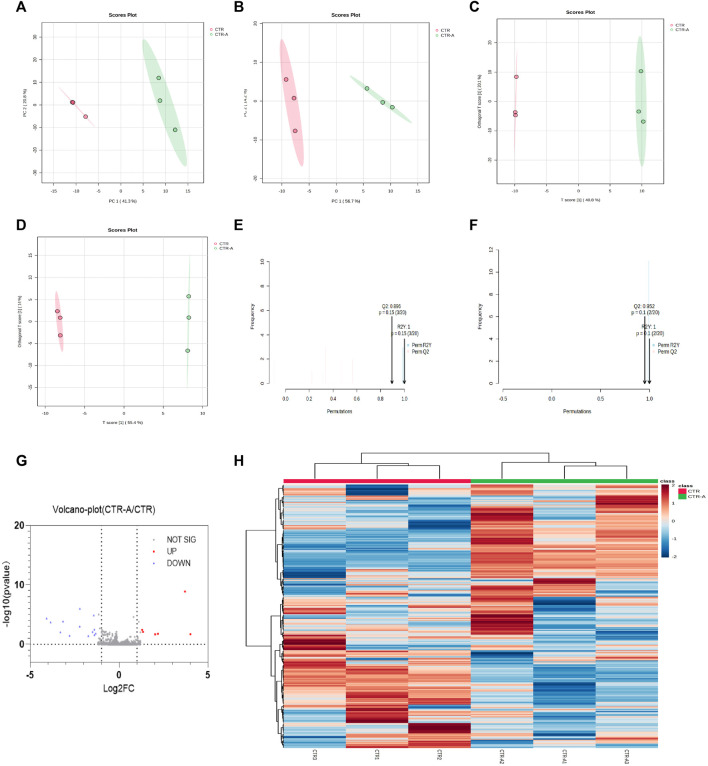
CPS-A regulates the expression of metabolites in the intestinal flora of normal mice. Score plots from the CTR, CTR + A for PCA and OPLS-DA. **(A)** ESI+, PCA analysis; **(B)** ESI-, PCA analysis; **(C)** ESI+, OPLS-DA analysis; **(D)** ESI-, OPLS-DA analysis, Permutations Plot analysis **(E,F)**, **(G)** volcano plot of CTR + A/CTR, **(H)** Heatmap analysis.

Intestinal flora plays a role in the transformation of CPS-A, and CPS-A often has a regulatory effect on intestinal flora derived metabolites. Through dimensionality reduction analysis of the data, Fecal metabolic profiles of normal mice (CTR) and mice treated with CPS-A *in vitro* (CTR + A) were analyzed using PCA in unsupervised mode and OPLS-DA in supervised mode. As shown in Figures 5A, B, fecal samples of the two groups of mice were obviously divided into two clusters under the positive and negative ion mode, indicating that the intervention of CPS-A had a significant disturbance on the metabolic level of mice. In order to better draw the metabolic profile, the OPLS-DA model in supervised mode is often applied for discriminant analysis of existing variables. (Figures 5C, D). In OPLS-DA mode, the two groups of samples were more significantly distinguished. Meanwhile, permutation test was performed to verify the reliability of this OPLS-DA model. (Figures 5E, F). Volcano plots showed that 6 metabolites are upregulated, and 15 metabolites were downregulated in CPS-A intervention group compared to the control group. (Figure 5G). The upregulated metabolites are Phenmetrazine, Atipamezole, N-Acetyl-D-quinovosamine and TXB2, while the downregulated metabolites include Pyridoxamine, Veronal, methyprylon and so on. Information about upregulated and downregulated metabolites is shown in Supplementary Table S1. In order to better characterize the changes at the metabolic level, Fold change (FC) ≥1.2 or ≤0.8 and *p*-value of Student’s t-test statistical analysis (*p* < 0.05) were used in this study. The differential metabolites in each group were found and analyzed by heat map clustering in Figure 5H.

### Changes of intestinal flora metabolism in colitis mice after CPS-A intervention

**FIGURE 6:**
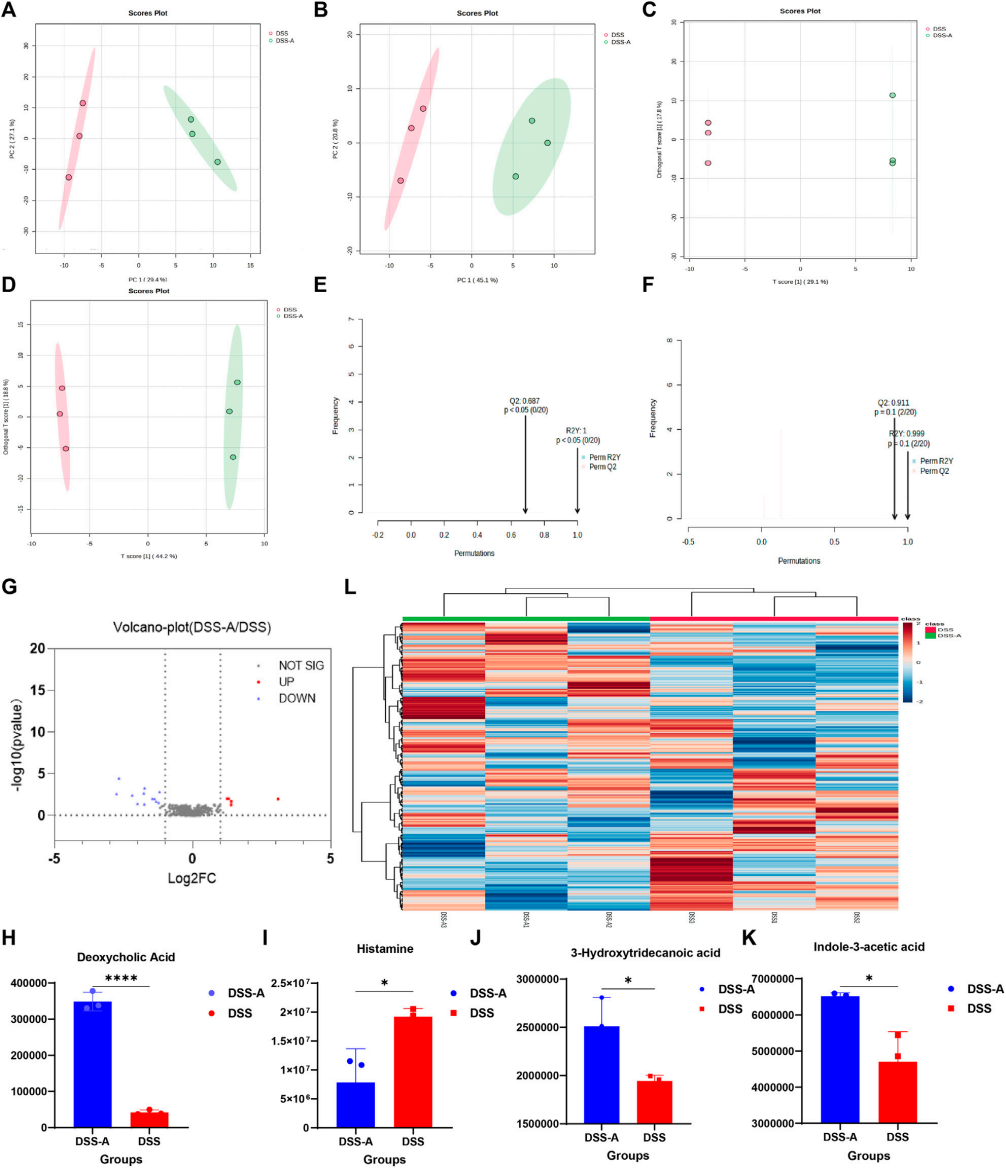
CPS-A regulates the expression of metabolites in the intestinal flora of colitis mice. Score plots from the DSS, DSS + A for PCA and OPLS-DA. **(A)** ESI+, PCA analysis; **(B)** ESI-, PCA analysis; **(C)** ESI+, OPLS-DA analysis; **(D)** ESI-, OPLS-DA analysis, Permutations Plot analysis **(E,F)**. **(G)** Volcano plot of DSS + A/DSS. Peak area comparison diagram of Deoxycholic Acid **(H)**, Histamine **(I)**, 3-Hydroxytridecanoic acid **(J)**, and Indole-3-acetic acid **(K)**. (**L)** Heatmap analysis. Values are expressed as mean ± SD. *p < 0.05, * *p < 0.01, ***p ≤ 0.001,****p ≤ 0.0001.

Compared to normal mice, the mice showed changes in the abundance and structure of their gut flora after developing colitis. Accordingly, under the action of CPS-A, intestinal microbiota related metabolites will also show differential changes. Multivariate analysis of the fecal metabolic profile of DSS mice (DSS) and mice treated with CPS-A *in vitro* (DSS + A) was performed using PCA in unsupervised mode and OPLS-DA in supervised mode. As shown in Figures 6A, B, the fecal samples of the two groups of mice were obviously divided into two clusters under the positive and negative ion mode, indicating that CPS-A significantly interferes with the metabolites of intestinal flora in colitis. The discriminant analysis of the existing variables by OPLS-DA in supervised mode found that the two groups of samples were more obviously divided into two clusters (Figures 6C, D). Permutation test showed the values of R2 and 02 are both higher than 0.5, indicating good model fitting accuracy. (Figures 6E, F). Volcano plots showed that 5 metabolites are upregulated, and 8 metabolites were downregulated in CPS-A intervention group compared to the DSS group. (Figure 6G), Specific information on upregulated and downregulated metabolites were shown in Supplementary Table S2. Compared with the DSS group, concentrations of Deoxycholic Acid, Histamine, 3-Hydroxytridecanoic acid, and Indole-3-acetic acid all increased significantly after *in vitro* administration of CPS-A, which could be found to be consistent with the beneficial effects of colitis. (Figures 6H–K). Fold change (FC) ≥1.2 or ≤0.8 and *p*-value of Student’s t-test statistical analysis (*p* < 0.05) were used to screen for differential metabolites. Heatmap clustering in Figure 6L demonstrated the difference in metabolites between the two groups.

### The difference of the metabolites of intestinal flora in normal mice and colitis mice by CPS-A intervention

**FIGURE 7:**
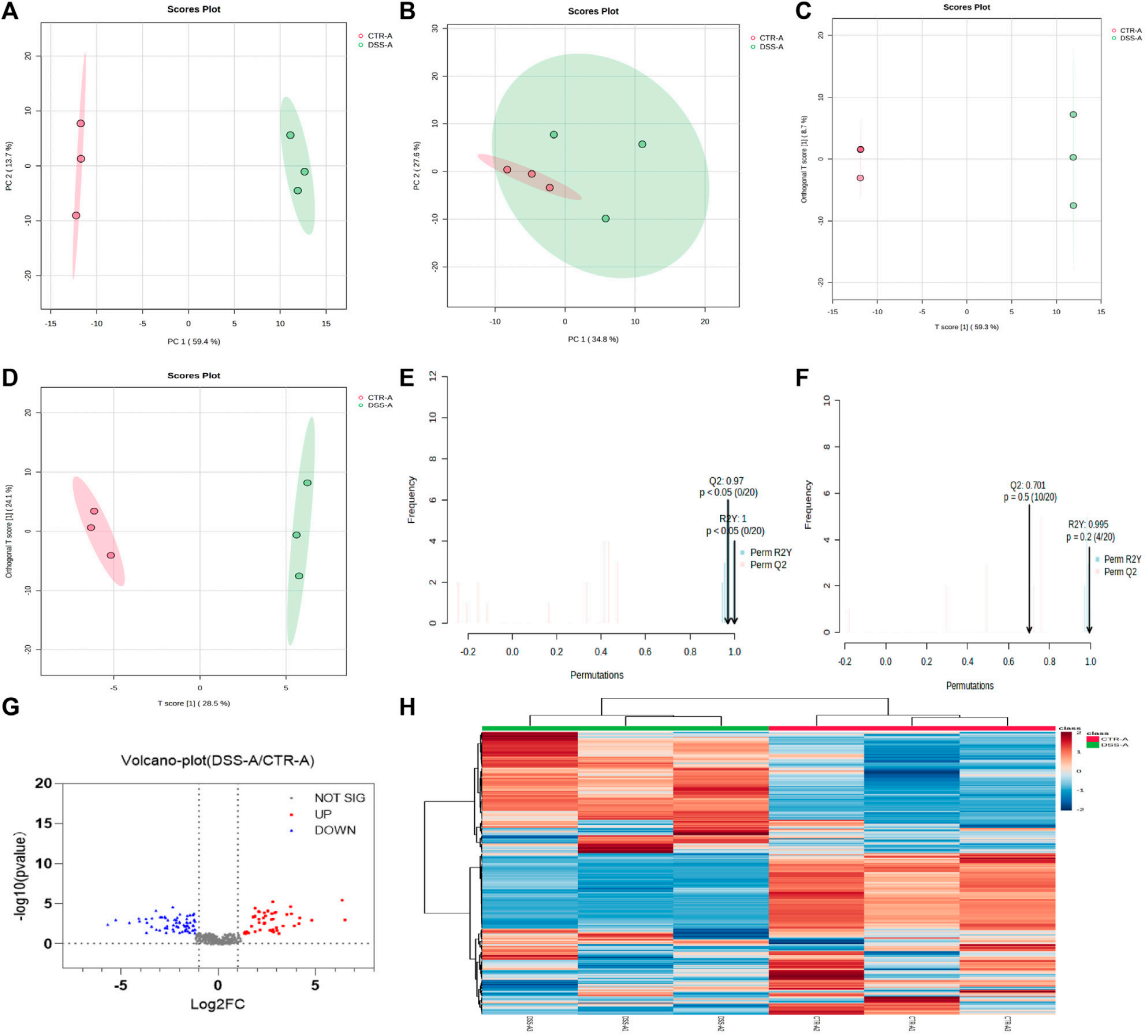
CPS-A regulates differential expression of intestinal microbiota metabolites in normal and colitis mice. Score plots from the CTR + A, DSS + A for PCA and OPLS-DA. **(A)** ESI+, PCA analysis; **(B)** ESI-, PCA analysis; **(C)** ESI+, OPLS-DA analysis; **(D)** ESI-, OPLS-DA analysis, Permutations Plot analysis **(E,F)**, **(G)** Volcano plot of CTR + A/DSS + A, **(H)** Heatmap analysis.

CPS-A has significant effects on intestinal flora metabolites of normal mice and colitis mice. Next, we analyzed the differences in the intervention of CPS-A on two different intestinal flora metabolites. A metabolic profile with PCA and OPLS-DA was obtained by employing the existing analytical method to evaluate the feces samples in ESI+ and ESI- modes. The PCA analysis score plots were displayed in Figures 7A, B. In ESI + mode, Both PCA models enabled the division of samples from the two groups into distinct blocks, demonstrating that the metabolites were significantly different between them. However, in ESI- mode, The PCA analysis score plots showed the CTR + A group was included under the module of the DSS + A group. In OPLS-DA mode, the two groups of samples were significantly distinguished. (Figures 7C, D). The results of permutation test showed that OPLS-DA had good predictive power. (Figures 7E, F). We further compared the metabolites of CTR + A group with DSS + A group, and found that CPS-A, after acting on intestinal microbes in colitis state, upregulated 45 metabolites and downregulated 65 metabolites. (Figure 7G). The upregulated metabolites are mainly 2-Hydroxyvaleric acid, 2-methylbutyrylcarnitine, 3,4-Dimethylbenzoic acid, acetyl proline, and 3,4-Dimethylbenzoic acid, while the downregulated metabolites mainly include 2-monolinolenin, 6-APA, amfonelic acid, and so on. Specific information of differential metabolites on metabolites was shown in Supplementary Table S3. To compare the changes in microbiota metabolites after CPS-A action in different disease models, these microbiota metabolites with statistical differences may be related to the therapeutic effect of the disease, and therefore will be the next target of interest. The heat map also clearly showed the difference in metabolites between the two groups. (Figure 7H). Under the action of CPS-A, the intestinal microbiota metabolites of normal mice and colitis mice showed more different changes.

### Enrichment analysis of metabolic pathway and spearman correlation

**FIGURE 8:**
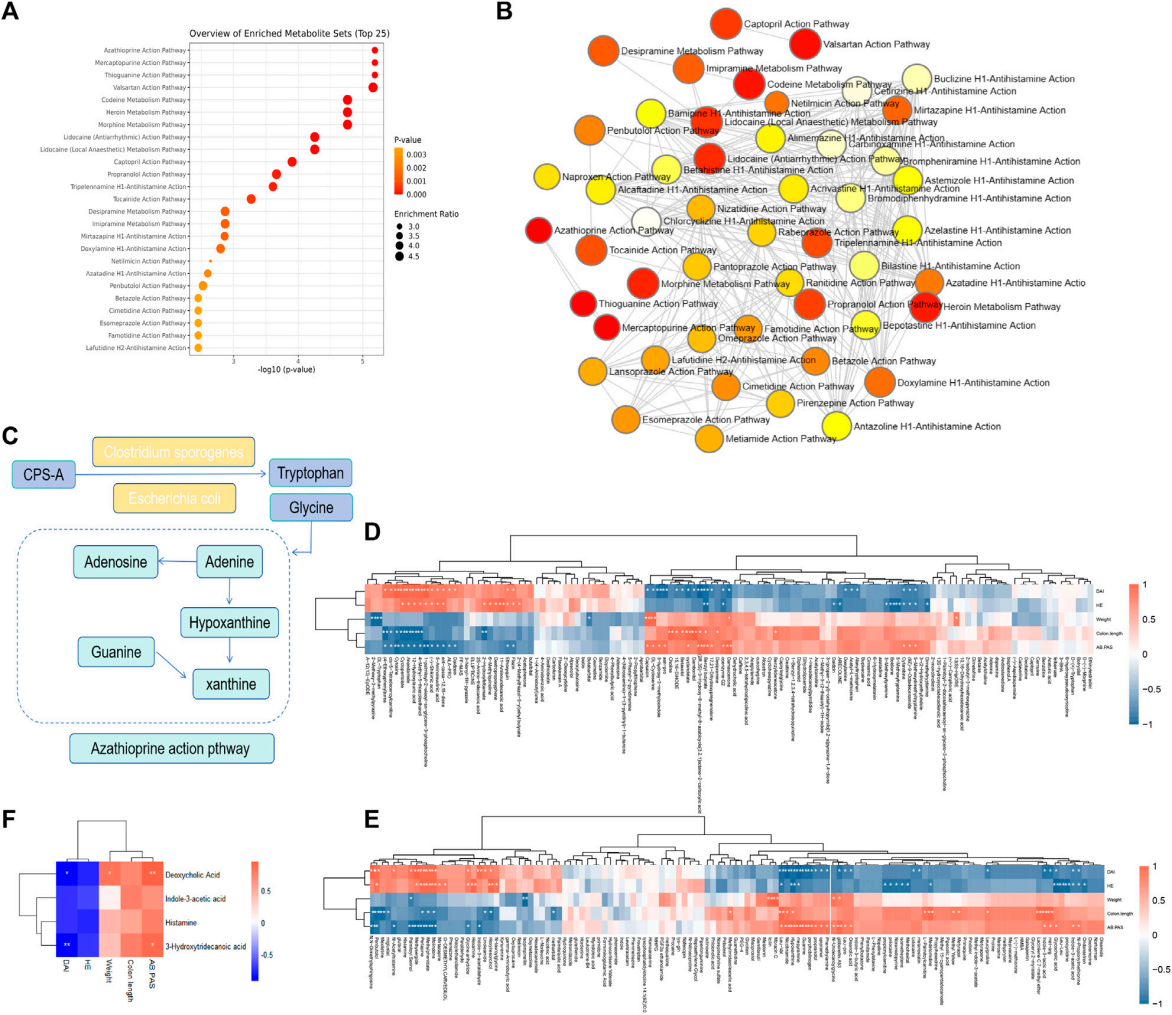
Enrichment analysis of metabolic pathway and Spearman correlation. **(A)** Enrichment analysis of metabolic pathway. **(B)** Path enrichment network diagram. **(C)** Diagram of the metabolic pathway regulated by CPS-A. **(D)** Spearman correlation heatmap between differential metabolites and colitis phenotype. **(E)** Spearman correlation heatmap between Upregulated or downregulated metabolites and colitis phenotypes. **(F)** Spearman correlation heatmap between key metabolites and colitis phenotypes.

From the above results, it can be found that CPS-A can regulate intestinal flora derived metabolites, and the regulatory effects of different intestinal flora are also significantly different. Next, Metabaalayst 5.0 software was used to analyze the metabolic pathways. It was found that the metabolic pathways mainly involved the metabolism of azathioprine and Mercaptopurine. (Figures 8A, B). As shown in Figure 8C, the schematic diagram of changes in major metabolites and metabolic pathways was used as a metabolic network. CPS-A affects the production of tryptophan and glycine metabolites through interaction with intestinal flora such as *Clostridium sporogeneses* and *Escherichia coli*, and then acts on purine metabolism. It could also be a way to benefit from colitis. Studies have shown that drugs can play an anti-colitis role by interacting with the gut flora and regulating related metabolites (Wu et al., 2020; Qu et al., 2022). Based on the above facts, we analyzed the correlation between the differential metabolites and colitis assessment indicators. As shown in Figures 8D, E, there was a strong correlation between most metabolites and colitis assessment indicators. We further analyzed the correlation between significantly elevated metabolites of CPS-A *in vitro* after treatment of colitis stool and body weight, DAI, colon length, HE, and AB-PAS in colitis mice. As shown in Figure 8F, there was a strong correlation between them. Importantly, Deoxycholic Acid was significantly positively correlated with body weight and AB-PAS and negatively correlated with DAI in colitis mice. 3-Hydroxytridecanoic acid was significantly positively correlated with AB-PAS and negatively correlated with DAI in colitis mice. This is consistent with literature reports (Zhao et al., 2016), and it can be speculated that CPS-A may also play a role through these metabolites when acting on colitis *in vivo*.

## Discussion

Saponins are a class of small amphiphilic compounds with high molecular weight. These substances were divided into triterpenoid saponins and steroid saponins according to the difference of aglycones (Niu et al., 2013). Due to the special structure of saponins, the bioavailability is low, and the residence time in the intestine is longer (Chen et al., 2017). Therefore, gut microbes are especially important for the biotransformation of saponins. The saponins are converted into smaller molecules by sugar hydrolysis reaction under the action of intestinal microorganisms, so as to improve bioavailability and enhance drug action. Deglycosylation is the main type of glycoside hydrolysis that occurs in the gut (Xu et al., 2017). Previous studies have found CPS-A does not undergo transformation in plasma but takes the form of desugar transformation in urine. The most important metabolic transformation is in feces, through desugar, dehydration, methylation, and other ways (Li et al., 2023). In our study, by incubating CPS-A with intestinal flora *in vitro*, it was found that CPS-A could be transformed into corresponding secondary metabolites through deglycosylation, methylation and dehydration under the action of intestinal flora. Among them, deglycosylation was the main method, and the abundance of deglycosylation products accounted for 92% of the total product abundance of CPS-A.

Altered microbiota and metabolites and transformed Chinese medicine metabolites may contribute to the control of disease progression. Previous studies have found that platycodon saponins were metabolized by sugar hydrolysis, aglycones dehydroxylation and acetylation under the action of human intestinal microorganisms (Ha et al., 2010). Ginsenoside Rb1 may be converted into compound K by gastrointestinal microbiota to enhance its pharmacological effects (Wan et al., 2016). It has also been found that lancemaside A is subjected to microbial action in the gastrointestinal tract and hydrolyzed to produce deglycosylated metabolites, thus producing pharmacodynamic activities (Komoto et al., 2010). Chen et al. found that intestinal flora converts saponin glycosyl-base bonds into potential bioactive metabolites ginsenoside F1, protopanaxatriol, ginsenoside RH2, Ginsenoside F1, Ginsenoside RH2, Ginsenoside F1 and Ginsenoside RH2. Ginsenoside compound K and protopanaxadiol, and these metabolites have stronger pharmacological activity (Chen et al., 2017).

Saponins interact closely with intestinal microbes, which also play a key role in converting into small molecule metabolites under the action of CPS-A. In this study, we constructed the fecal incubation system of CPS-A with normal mice and colitis mice respectively *in vitro* and found that the metabolites of CPS-A acting on different intestinal microflora were also different. Among them, the differential metabolites were found to be involved in azathioprine and mercaptopurine metabolic pathways through enrichment pathways. However, amino acids such as glycine and tryptophan participated in this pathway (Yachida et al., 2019). This may be due to the action of CPS-A through small molecule metabolites metabolized by other strains such as *C. sporogeneses* and *E. coli.* Some studies have shown that there are certain differences in metabolic capacity between different strains. For example, several *Bacteroides* and *Clostridium* can produce indole lactic acid, while *Bifidobacterium* produces indole lactic acid (Daliri et al., 2017). Studies have shown that berberine can play an anti-colitis role by regulating related metabolites through interaction with intestinal flora (Jing et al., 2021). Importantly, Protopanaxatriol saponin can regulate a variety of differentiated metabolites and metabolic pathways (Wu et al., 2022). Studies have also shown that spermidine can inhibit the activation of F4/80 macrophages and T cells, reduce the expression of pro-inflammatory cytokines and the phosphorylation of NF-κB and MAPK, and improve colitis through the above pathways (Ma et al., 2021). At the same time, our study was conducted *in vitro* by incubating CPS-A with colitis mouse feces. Intestinal flora metabolites Deoxycholic acid, Histamine, 3-Hydroxytridecanoic acid, and Indole-3-acetic acid changed significantly after the operation of CPS-A. Studies have shown that in inflammable UC patients, dysbiosis leads to a deficiency of deoxycholic acid, which exacerbates the inflammatory state in the gut (Sinha et al., 2020). Importantly, FBTP can reshape the gut microbiome and promote the conversion of tryptophan to indole-3-acetic acid by the microbes, which subsequently leads to colitis protection by enhancing the expression of IL-22 and tight-linking proteins in the colon (Zhang et al., 2023). These metabolites are beneficial to relieve colon inflammation. Preliminary found that the *in vivo* action of CPS-A on colon cancer mainly involves amino acid metabolism and purine metabolism, and our *in vitro* action on colitis also mainly involves these two metabolisms (Li et al., 2023). Colitis is known to have a risk of progressing to colon cancer (Shah and Itzkowitz, 2022). This further confirms our results.

The main innovation of this study is to construct a reaction system between saponins such as CPS-A and intestinal flora *in vitro*, which is more convenient for researchers to understand how they interact and discover new bioactive ingredients. However, whether these new active ingredients have antioxidant, anti-inflammatory, antibacterial and other properties still needs further research. It can also be more targeted to guide intestinal flora regulation strategies. Meanwhile, there are some shortcomings in this study. First, we only focused on the interaction between CPS-A and the overall intestinal flora, and did not deeply explore the relationship between a particular bacterium and CPS-A. Secondly, this paper only selected colitis model specimens with a high cancer rate for study, which limited the significance of the disease. Third, the products after interaction need to be further verified.

## Conclusion

In this study, based on metabolomics, the reaction system of intestinal flora *in vitro* was constructed. The results of *in vitro* experiments indicate that intestinal microorganisms can mediate the biotransformation of CPS-A and metabolize it into corresponding deglycosylation products, thereby promoting its drug effect. Not only that, CPS-A can also promote metabolites such as Deoxycholic acid, Histamine, 3-Hydroxytridecanoic acid, and Indole-3-acetic acid in the intestinal microbiota of mice with colitis. This may result in anti-colitis effects. CPS-A mainly involved in metabolic pathways such as azathioprine and mercaptopurine, which may also have beneficial or adverse effects. The study of the interaction of CPS-A with the microbiota in this study provides new insights into traditional herbs with poor oral bioavailability. And the regulatory effect of CPS-A on the metabolites of intestinal flora in colitis mice was also found. It laid the foundation for the mechanism of the saponin action in colitis mice. The interaction between Chinese medicine and intestinal flora affects the ecological balance and health of human body. The efficacy of TCM in treating related diseases by regulating intestinal flora and its metabolites has been recognized more and more clinically. The intestinal flora can transform the active components of traditional Chinese medicine into metabolites with high bioavailability and pharmacological activity through biotransformation. It will be a new idea to search for effective substances in intestinal flora metabolism of Chinese medicine transfer.

## Data Availability

The original contributions presented in the study are included in the article/Supplementary Material, further inquiries can be directed to the corresponding authors.

## Supplementary material

SUPPLEMENTARY FIGURE S1 Metabolite map of CPS-A under the action of intestinal microbiota in vitro.
