# Supplementary material for: In-depth LC-MS and in-vitro studies of a triterpenoid saponin capilliposide-A metabolism modulation in gut microbiota of mice
Source: Front Pharmacol. 2024 Mar 14;15:1361643. doi: 10.3389/fphar.2024.1361643 (PMC10973126; doi:10.3389/fphar.2024.1361643)
Supplement: Supplementary file 1 [file Table1.pdf]

Table S1: Specific information on up-regulated and down-regulated metabolites between the CTR and CTR+A groups.

Description	change	Formula	m/z	log2FC	Anova (p)	MassError (ppm)	Scan mode
Phenmetrazine	UP	C11 H15 N O	178.12259	1.34	0.007838716	-0.28	ESI+
Atipamezole	UP	C14 H16 N2	213.1386	4.01	0.020846225	-0.1	ESI+
N-Acetyl-D-quinovosamine	UP	C8 H15 N O5	206.10134	2.02	0.021279744	-4.67	ESI+
TxB2	UP	C20 H34 O6	371.24304	1.29	0.003450624	0.61	ESI+
Pyridoxamine	Down	C8 H12 N2 O2	169.09711	-4.08	0.000043012	-0.25	ESI+
Veronal	Down	C8 H12 N2 O3	185.09203	-1.32	0.014914178	-0.2	ESI+
methyprylon	Down	C10 H17 N O2	184.1331	-1.5	0.034399544	-1.5	ESI+
R-Cathinone	Down	C9 H11 N O	150.09138	-2.8	0.034399544	0.26	ESI+
Aprobarbital	Down	C10 H14 N2 O3	211.10777	-1.43	0.0000130612	0.25	ESI+
Uracil	Down	C4 H4 N2 O2	113.03445	-0.96	0.00019335	-0.96	ESI+
Valylproline	Down	C10 H18 N2 O3	215.13894	-2.22	0.000980827	-0.39	ESI+
Nitrosoguvacoline	Down	C10 H18 N2 O3	171.07639	-2.21	0.000000985883	-0.17	ESI+
Nicotinic acid	Down	C6 H5 N O2	124.03925	-1.4	0.003211935	-0.21	ESI+
primidone	Down	C12 H14 N2 O2	219.11269	-3.31	0.009481967	-0.52	ESI+
6-Hydroxymelatonin	Down	C13 H16 N2 O3	249.12339	-3.16	0.00013323	0.09	ESI+

Table S2: Specific information on up-regulated and down-regulated metabolites between the DSS and DSS+A groups.

Description	change	Formula	m/z	log2FC	Anova (p)	MassError (ppm)	Scan mode
3-Hydroxytridecanoic acid	UP	C13 H26 O3	229.18088	1.393368778	0.019968757	-0.16	ESI-
Deoxycholic Acid	UP	C24 H40 O4	391.28546	3.087266685	0.010053423	0.27	ESI-
Pivagabine	UP	C9 H17 N O3	188.12807	1.291996844	0.009381925	-0.28	ESI+
feruloylserotonin	Down	C20 H20 N2 O4	351.13464	-2.004233678	0.036232372	-1.1	ESI+
Histamine	Down	C5 H9 N3	112.08678	-1.387728548	0.0096784	-1.28	ESI+
imicyafos	Down	C11 H21 N4 O2 P S	303.10458	-1.327174843	0.020321938	-1.39	ESI-
Methylphenidate	Down	C14 H19 N O2	234.1488	-1.46518017	0.009370532	-0.26	ESI+
Oxagrelate	Down	C14 H16 N2 O4	277.11822	-2.199007465	0.003712257	-0.22	ESI+
Paracetamol	Down	C11 H14 N2 O4 S	271.07498	-1.235999706	0.028334207	-0.94	ESI+
Pyridoxine	Down	C8 H11 N O3	170.08113	-1.749657058	0.00051481	-0.24	ESI+
Ritalinic acid	Down	C13 H17 N O2	220.13309	-2.764594538	0.002483914	-0.53	ESI+
Thymidine	Down	C10 H14 N2 O5	287.08859	-2.676572819	0.0000345	0.33	ESI-
Tiglylcarnitine	Down	C12 H21 N O4	244.15418	-1.756035461	0.043103138	-0.65	ESI+
Vorinostat	Down	C14 H20 N2 O3	265.15449	-1.777553654	0.002048178	-0.68	ESI+

Table S3: Specific information on up-regulated and down-regulated metabolites between the CTR+A and DSS+A groups.

Description	change	Formula	m/z	log2FC	Anova (p)	MassError (ppm)	Scan mode
2-Hydroxyvaleric acid	UP	C5 H10 O3	117.05585	2.737017715	0.000910633	1.1	ESI-
2-methylbutyrylcarnitine	UP	C12 H23 N O4	246.16998	4.138165076	0.002906832	-0.01	ESI+
3,4-Dimethylbenzoic acid	UP	C9 H10 O2	149.06086	2.088544419	0.000824412	0.4	ESI+
3-Phenylpropyl hydrogen sulfate	UP	C9 H12 O4 S	215.03849	1.700581787	0.006383273	0.64	ESI-
4-Hydroxybutyric acid	UP	C4 H8 O3	103.04022	2.653594874	0.019832625	1.47	ESI+
6-Hydroxycaproic acid	UP	C6 H12 O3	131.07144	1.337706599	0.046166996	0.53	ESI-
acetyl proline	UP	C7 H11 N O3	158.08134	2.354264874	0.002767047	0.34	ESI+
Biotin l-Sulfoxide	UP	C10 H16 N2 O4 S	259.07643	3.901452515	0.005397991	2.43	ESI-
Brivaracetam	UP	C11 H20 N2 O2	213.15969	2.891549774	0.000135973	-0.31	ESI+
Ceceline	UP	C19 H16 N2 O2	305.12851	2.81303551	0.000781334	0.19	ESI+
Docosatetraenoylethanolamide	UP	C24 H41 N O2	376.32101	2.361263563	0.000133566	0.01	ESI+
Fenspiride	UP	C15 H20 N2 O2	261.1596	3.316587242	0.005699556	-0.59	ESI+
hypaphorine	UP	C14 H18 N2 O2	247.14407	2.948668268	0.000115109	-0.15	ESI+
Hypoxanthine	UP	C5 H4 N4 O	137.04583	2.318573394	0.000587629	0.4	ESI+
Indole-3-acetic acid	UP	C10 H9 N O2	176.07053	1.35604039	0.047675293	-0.42	ESI+
Isoprenaline	UP	C11 H17 N O3	212.12817	2.139156857	0.017723503	0.23	ESI+
Levulinic acid	UP	C5 H9 N O3	132.06551	2.077403893	0.0000899	-0.06	ESI+
N-Acetylhistamine	UP	C7 H11 N3 O	154.0975	6.511216926	0.001010686	0.07	ESI+
n-Butyl lactate	UP	C7 H14 O3	145.08712	3.759929125	0.000176236	0.72	ESI-
Paeonilactone C	UP	C17 H18 O6	317.10405	2.82898513	0.032430662	3.1	ESI-
pentobarbital	UP	C11 H18 N2 O3	227.13894	2.750636177	0.023284009	-0.33	ESI-
Phenylethyl alcohol	UP	C8 H10 O	121.06601	2.746510235	0.000161092	1.03	ESI-
Pibutidine	UP	C19 H24 N4 O3	355.17765	3.005692408	0.021889887	0.24	ESI+
Piracetam	UP	C6 H10 N2 O2	141.06698	2.798543948	0.00000573	0.19	ESI+
propionylcarnitine	UP	C10 H19 N O4	218.13859	1.883319441	0.000360197	-0.39	ESI+
Sulfosalicylic Acid	UP	C7 H6 O6 S	216.98051	4.792288019	0.001094768	-3.31	ESI-
trimethadione	UP	C6 H9 N O3	142.05105	2.375920085	0.00233048	0.58	ESI-
Viloxazine	UP	C13 H19 N O3	236.12922	1.46356684	0.030800945	0.01	ESI-
12-Hydroxylauric acid	Down	C12 H24 O3	217.17981	-1.247030964	0.000938826	-0.05	ESI+
16-Hydroxyhexadecanoic acid	Down	C16 H32 O3	271.22795	-1.324923137	0.020786833	0.32	ESI-
2-monolinolenin	Down	C21 H36 O4	353.26875	-1.481814519	0.037554222	0.26	ESI+
6-APA	Down	C8 H12 N2 O3 S	215.04938	-3.434117968	0.0069634	-0.67	ESI-
amfonelic acid	Down	C18 H16 N2 O3	309.12346	-1.657492634	0.013195546	0.31	ESI+
Anatalline	Down	C15 H17 N3	238.13489	-2.689753097	0.040413433	-0.34	ESI-
Apomorphine	Down	C17 H17 N O2	266.11872	-2.118734683	0.000520049	0.26	ESI-
Brassicanal A	Down	C10 H9 N O S	190.0333	-1.700904891	0.02483872	0.46	ESI-

Carbofuran	Down	C12 H15 N O3	222.11241	-1.516538335	0.00926097	-0.25	ESI+
Cetilistat	Down	C25 H39 N O3	400.28559	-2.993514435	0.000440668	-0.33	ESI-
cyclopeptine	Down	C17 H16 N2 O2	281.12847	-5.69199434	0.003845612	0.07	ESI+
dacisteine	Down	C7 H11 N O4 S	204.03373	-1.30114552	0.004500481	1.96	ESI-
diphenyldisulfide	Down	C12 H10 S2	217.01519	-2.177740118	0.00104544	0.34	ESI-
DL-Phenylalanine	Down	C9 H11 N O2	164.07179	-1.585181933	0.005357137	0.53	ESI-
Leucine	Down	C6 H13 N O2	130.0875	-1.294481539	0.00542709	1.12	ESI-
imazamethabenz-methyl	Down	C16 H20 N2 O3	289.15482	-1.784251226	0.004122001	0.51	ESI+
Leu-Leu	Down	C12 H24 N2 O3	245.18577	-1.213025607	0.044078534	-0.86	ESI+
Leu-Val	Down	C11 H22 N2 O3	231.17026	-1.250804136	0.001145837	-0.23	ESI+
Linoleic Acid	Down	C18 H32 O2	279.23304	-1.596474016	0.002322817	0.3	ESI-
Maleic acid	Down	C4 H4 O4	175.0249	-1.863619215	0.029179323	1.08	ESI-
Mangostanol	Down	C24 H26 O7	425.16139	-1.25243705	0.017983481	1.92	ESI-
Methylphenidate	Down	C14 H19 N O2	234.1488	-2.175221109	0.002167668	-0.26	ESI+
N-Acetyl-L-methionine	Down	C7 H13 N O3 S	190.05444	-1.992756622	0.039131971	0.56	ESI-
Sophoramine	Down	C15 H20 N2 O	245.16463	-2.742212672	0.010372276	-0.87	ESI+
Valylvaline	Down	C10 H20 N2 O3	217.15463	-1.204354926	0.001579909	-0.18	ESI+
tybamate	Down	C13 H26 N2 O4	273.18209	-2.313071017	0.004539432	0.35	ESI-
Vorinostat	Down	C14 H20 N2 O3	265.15429	-2.756996266	0.000411287	-1.44	ESI+
Z-Gly-Pro-Leu-Gly-Pro	Down	C28 H39 N5 O8	572.27278	-2.338325727	0.014695136	0.33	ESI+
