# Supplementary figures and images for: In-depth LC-MS and in-vitro studies of a triterpenoid saponin capilliposide-A metabolism modulation in gut microbiota of mice
Source: Front Pharmacol. 2024 Mar 14;15:1361643. doi: 10.3389/fphar.2024.1361643 (PMC10973126; doi:10.3389/fphar.2024.1361643)

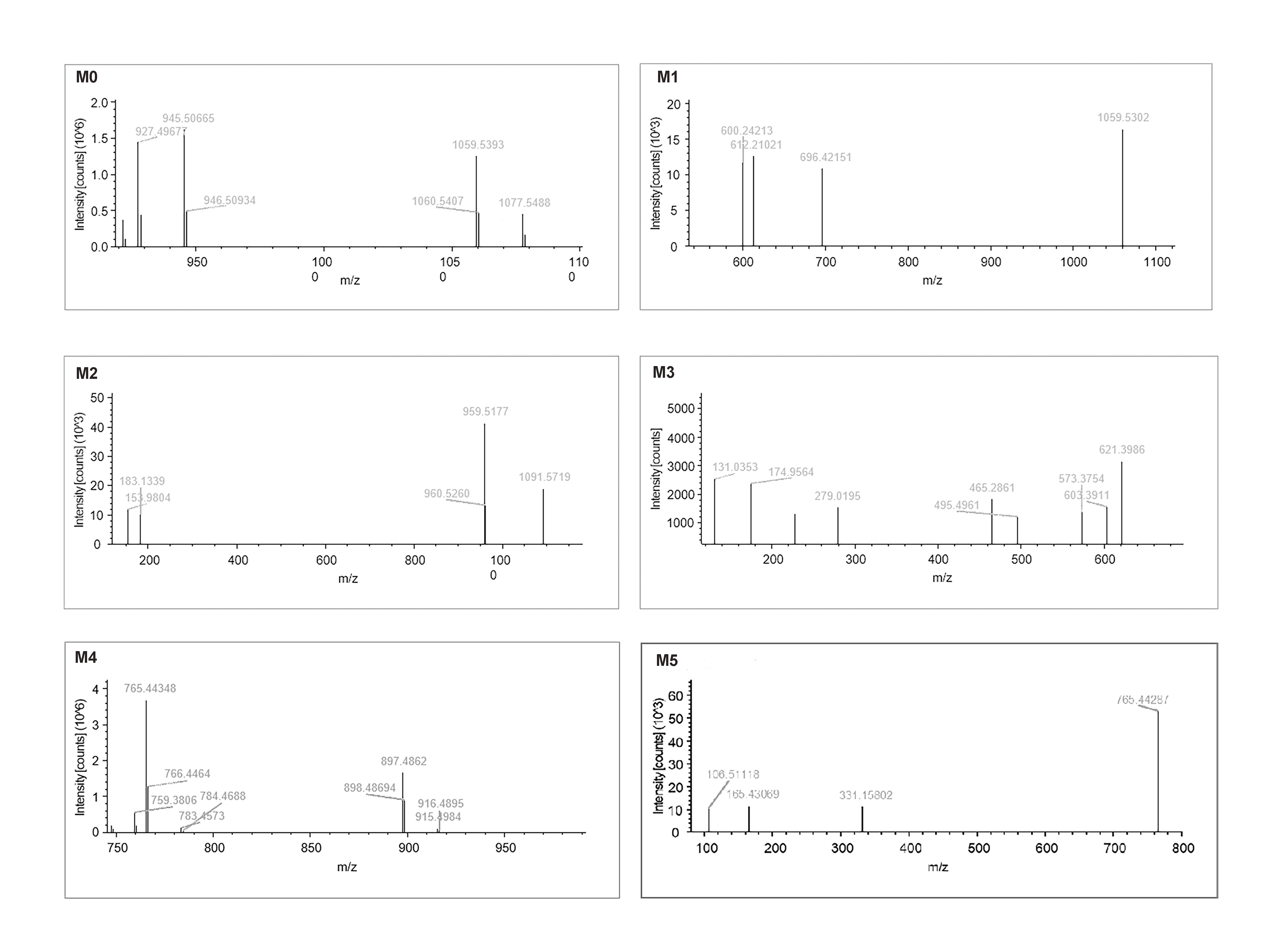

Supplement: Supplementary file 2 [file Image1.JPEG]
